# Alginate and Nanocellulose Dressings With Extract From Salmon Roe Reduce Inflammation and Accelerate Healing of Porcine Burn Wounds

**DOI:** 10.1093/jbcr/irad006

**Published:** 2023-01-14

**Authors:** Karin M Gilljam, Patrik Stenlund, Simon Standoft, Sisse Bindslev Andersen, Kari Kaaber, Henrik Lund, Karl R K Bryn

**Affiliations:** Regenics AS, Gaustadalléen 21, N-0349 Oslo, Norway; RISE Research Institutes of Sweden AB, Department of Methodology, Textile and Medical Technology, Arvid Wallgrens backe 20, SE-413 46 Göteborg, Sweden; RISE Research Institutes of Sweden AB, Department of Methodology, Textile and Medical Technology, Arvid Wallgrens backe 20, SE-413 46 Göteborg, Sweden; Scantox A/S, Department of Toxicology Science, Hestehavevej, 36A, 4623, Lille Skensved, Denmark; Scantox A/S, Department of Toxicology Science, Hestehavevej, 36A, 4623, Lille Skensved, Denmark; Regenics AS, Gaustadalléen 21, N-0349 Oslo, Norway; Regenics AS, Gaustadalléen 21, N-0349 Oslo, Norway

## Abstract

Partial-thickness thermal burn wounds are characterized by a prolonged inflammatory response, oxidative stress, tissue damage, and secondary necrosis. An optimal dressing for burn wounds would reduce inflammation and oxidative stress while providing a moist, absorbent, and protective cover. We have developed an extract from unfertilized salmon roe containing components with potential anti-inflammatory and antioxidative properties, called HTX. HTX has been combined with alginate from brown algae and nanocellulose from tunicates, and 3D printed into a solid hydrogel wound dressing called Collex. Here, Collex was tested on partial thickness burn wounds in Göttingen minipigs compared to Jelonet, and a variant of Collex without HTX. We found that dermal treatment of burn wounds with Collex resulted in accelerated healing at a majority of measured points over 23 days, compared to treatment with Jelonet. In comparison to Collex without HTX, Collex enhanced healing in the first week after trauma where wound progression was pronounced. Notably, Collex reduced the inflammatory response in the early post-injury phase. The anti-inflammatory response of Collex was investigated in more detail on activated M1 macrophages. We found that Collex, as well as HTX alone, significantly reduced the secretion of pro-inflammatory interleukin-1β as well as intracellular levels of oxidative stress. The results from this study indicate that Collex is a potent dressing for the treatment of burn wounds, with the anti-inflammatory effect of HTX beneficial in the initial phase, and the moist qualities of the hydrogel favorable both in the initial and the proceeding proliferative phase of wound healing.

## INTRODUCTION

Burn injuries are traumatic, painful, and associated with slow skin recovery and massive scarring. Healing of burn wounds follows the same steps as all wounds, but differ by the severity and duration of the inflammatory phase.^1^ The inflammatory phase, partly orchestrated by macrophages, is vital, but in burn wounds, the pro-inflammatory M1 macrophage phenotype is predominant, causing an excessive and prolonged inflammatory response.^2,3^ Reactive oxygen species (ROS) play a pivotal role in several wound healing processes, however, burn wounds generate excessive ROS, called oxidative stress.^4^ Inflammation and ROS mutually trigger each other, and together contribute to secondary necrosis, delayed wound healing, and hypertrophic scarring.^2,3,5–8^

Current standard-of-care dressings for partial-thickness burn wounds aim to cover and protect the wound surface from infection, maintain a moist environment, and reduce discomfort for the patient. In Norwegian hospitals, burn wounds are initially covered by the tulle gras dressing Jelonet, typically replaced by foam dressings with anti-infective properties.^9^ Alginate and nanocellulose hydrogels are currently used in wound treatment because they have good biocompatibility, ensure a physiologically moist environment, have liquid absorption capacity, reduce the risk of bacterial infection, and facilitate wound healing.^10,11^ In addition, they have the ability to contain and release drugs, and in current available dressings, these biomaterials are combined with anti-infective agents.^12–15^

While dressings with anti-infective agents are widely available, dressings with incorporated anti-inflammatory and antioxidant activity are being investigated.^16–18^ However, to the best of our knowledge, such dressings are not yet in regular that is non-research, clinical use. We aimed to develop a novel and improved dressing for the treatment of burn wounds by targeting the inflammatory phase while the standard mechanical qualities for moist wound healing was maintained. To this end, we combined the qualities of alginate and nanocellulose hydrogels with potential anti-inflammatory and anti-oxidative qualities of extract from unfertilized salmon roe, Heat-treated X (HTX). In this study, we have tested Collex, a 3D printed, solid, porous, hydrogel dressing made up from all-marine resources: Alginate from brown algae, nanocellulose from tunicates, and HTX from unfertilized salmon roe. HTX is a sterile extract purified from unfertilized salmon roe consisting of proteins (predominantly the egg yolk lipoprotein Vitellogenin), fatty acids (mainly unsaturated), DNA, and trace metals (mainly zinc) (unpublished data). Both Vitellogenin, unsaturated fatty acids and zinc are reported to reduce oxidative stress.^19–21^ Moreover, the high content of fatty acids in HTX is believed to enhance the moisturizing effect of Collex.^22^ Here, the safety and efficacy of Collex are tested on partial-thickness burn wounds in minipigs.

## METHODS

### Ethical Considerations

All animal experimentation was carried out by Scantox A/S, under a license approved by the National Animal Experiments Inspectorate under the Ministry of Food, Agriculture and Fisheries of Denmark, in accordance with the Guidance for Industry and Food and Drug Administration Staff (2020). This is in compliance with the OECD Principles of Good Laboratory Practice (GLP). When appropriate, in vitro assays on human cell lines were used to supplement data obtained from animal experimentation, reducing the need for large wounds and/or a higher number of animals.

### Production of Collex and Collex Without HTX

The ink for the production of Collex contains Alginate (2% (w/w), MVG, NovaMatrix AS), Nanocellulose supplemented with 4.6% Mannitol (1.7% (w/w), OceanTunicell AS), HTX (12% (v/w), Regenics AS), NaCl (0.9% (w/v) Fresenius Kabi), CaCl_2_ (0.02 M, SigmaAldrich). Approximately 200 µl of this ink (ca. 200 mg) was 3D printed using a fourth Gen 3D Bioplotter (EnvisionTEC, Gladbeck, Germany) into four layers of an interlaced 90-degree continuous grid pattern measuring 16 × 16 mm. The patch was crosslinked with 0.02M CaCl_2_ solution supplemented with saline (0.9% NaCl) and HTX (12%). Collex was kept in a polyethylene/aluminum sachet with an argon atmosphere and stored in a fridge prior to analysis. Production of Collex without HTX was identical to Collex, except HTX was replaced by saline (0.9% NaCl, Fresenius Kabi) to isolate the potential effects of HTX on healing of the burn wounds.

### Endotoxin Assay

The Endotoxin levels were tested in five Collex dressings of sizes between 190 and 197 mg. Collex was extracted in 0.9% NaCl in a total volume of 2 ml (1:10 dilution), incubated for 1 hour at 37℃, at 50 rpm. The tubes (containing dressing and extract) were then centrifuged for 30 minutes at 4000 rpm. Part of the extract (1 ml) at 1:10 ratio was transferred into the new tube. Subsequently, the extract was further diluted 1:100 (0.1 ml of extract + 0.9 ml of water (Biowest)), and the endotoxin levels tested by PyroGene™ kit (Lonza, #50-658U) and the WinKQCL™ Endotoxin Detection & Analysis Software. The analysis was performed according to the European Pharmacopoeia 2.6.32: Test for bacterial endotoxins using recombinant factor C.

### Animals: Housing, Anesthesia, and Pain treatment

The study included three female Göttingen minipigs from Ellegaard Göttingen Minipigs A/S. The housing of the animals was in accordance with EU Directive 2010/63/EU of September 22, 2010 on the protection of animals used for scientific purposes. On the day of wounding, anesthesia was achieved by an intramuscular injection in the neck (1.0 mL/10 kg) of a mixture of Zoletil 50®Vet., Virbac, France. Thereafter, the animals were prepared for surgery, intubated, and the anesthesia maintained by isoflurane.

For treatment of post-surgical pain, the animals were given a transdermal dressing with Fentanyl (75 µg/hour) for up to 72 hours from the day before surgery. In relation to the wounding procedure, the animals were given an intramuscular injection of methadone. In addition, the animals were given an intramuscular injection of meloxicam on the day of wounding (Day 1). For the following 2 days, treatment with meloxicamoral suspension continued once daily by oral administration. During the first week following wounding, the animals received an intramuscular injection of buprenorphine prior to collection of biopsies (Days 4 and 10), dosing, and change of bandage (Days 4, 6, and 10). All dosing and dressing changes were performed under general anesthesia.

On the day of necropsy (Day 23), the animals were weighed, examined externally, and anaesthetized by an intramuscular injection of a mixture of Zoletil 50 Vet., Virbac, France. The animals were terminated by exsanguination and were not fasted prior to necropsy.

### Burn Procedure

**Figure 1.**
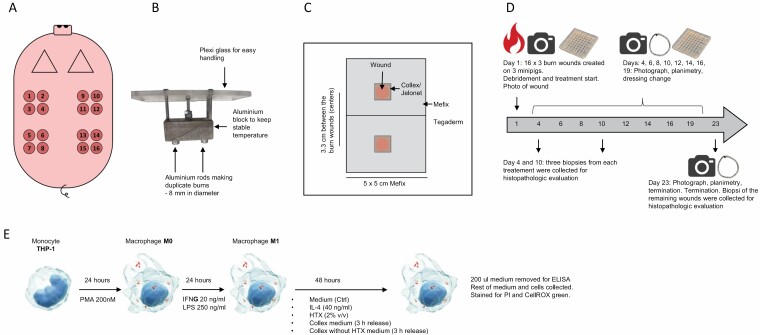
Methods illustration. (A) Illustration of burn wounds distribution on the back of the pig, duplicate burns, eight burns on each side of the spine. The representative treatments were placed in such a way that all animals received all treatments, and the distribution close to the head and close to the tail was equal for all treatments. (B) Photo of custom-made burn device used to create partial thickness burns on the pigs. Aluminum rods (making duplicate burns) are directly connected to an aluminum block to ensure stable temperature. The block is attached to a heat-stable Plexiglas plate for easy handling. (C) Illustration of the dressings used on the minipigs. Orange circles illustrate the burn wounds. Because Collex, Collex without HTX and Jelonet are non-adherent dressings they were fixed with Mefix. Tegaderm was included to ensure that the dressings were kept in place the whole time during the experiment. (D) Schematic illustration of the timeline in the minipig burn study. Numbers on the arrow indicate days. (E) Schematic illustration of method used for differentiation of THP-1 monocytes into macrophages, M0 and M1.

The wounding procedure was performed while the animals were under anesthesia. The dorso-lateral area of both sides of the back of the animal were clipped and further shaved using an electric shaver/trimmer, washed with soap and water, and rinsed with gaze soaked in sterile water. The area was disinfected with 70% ethanol and 5% iodine ethanol. Prior to initiation of the animal experiment, different temperatures and burn exposure times were tested on a surplus minipig. The wounds were followed for 2 days, and optimal time and temperature was determined based on macroscopic evaluation by veterinarian. On Day 1, 16 circular partial-thickness burn wounds (8 mm in diameter) were established on the back of each animal, eight on each side of the spine (Figure 1A). The burns were created with a custom-made aluminum block consisting of two cylinders (diameter 8 mm, Figure 1B). The aluminum block was attached by screws to an acrylic plate for easier handling, with the assembly weighing approximately 111 g. The aluminum block was initially preheated in boiling water. The heated aluminum block was wiped dry. Immediately after, the heated aluminum block was placed on the animal with the two cylinders directly in contact with the skin surface for a period of 25 ± 5 seconds.

### Dressings and Dressing Changes

Production of Collex and Collex without HTX is described above. Jelonet non-medicated tulle gras dressing (Smith and Nephew) was included as a non-active non-adherent dressing. Jelonet was cut in squares with the size matching Collex and Collex without HTX (1.6 × 1.6 cm). All three dressings are non-adherent and were fixed by Mefix from Mölnlycke (5 × 5 cm) and Tegaderm from 3M (10 × 10 cm) as outlined in Figure 1C. On top of these supportive dressings, Fixumull from BSN medical Wound Care was used and a netlike body stocking from BSN medical Wound Care was attached to a neck collar.

The dressings were changed at Days 4, 6, 8, 10, 12, 14, 16, and 19. At each dressing change, and at Day 23, the wounds were evaluated macroscopically by planimetric drawings. Photographs were taken for illustrative purposes. At Days 4 and 10, three wounds from each treatment group were biopsied and terminated. At the end of the experiments, remaining wounds were biopsied and send for histopathologic evaluation. The timeline is illustrated in Figure 1D.

### Processing and Microscopic Examination

On Days 4 and 10, the collection of biopsies took place while the animals were under anesthesia due to dressing change (Figure 1D). The biopsies were collected by an 8 mm punch biopsy and included tissue from the center and edge of the wound as well as part of unaffected skin surrounding the wound. At necropsy, all remaining wounds were cut free as a block (approximately 1 cm margin around the wound and if possible 0.5 cm margin to the bottom) separated from skeletal muscle tissue. All samples were fixed in phosphate-buffered neutral 4% formaldehyde. After fixation, representative specimens from the tissues specified for microscopic examination were trimmed and processed. The specimens were embedded in paraffin and cut at a nominal thickness of approximately 5 µm. The slides were stained with hematoxylin and eosin and examined by a Scantox A/S study pathologist. Slides were evaluated in accordance with ISO 10993-6: 2016. The scoring system was semi-quantitative, and microscopic evaluation was performed using a light microscope with an eyepiece-mounted grid to aid in the count of features of the histological sections. The depth of the burn wound was evaluated based upon damage to the epidermis and dermis and damage to the pilo-sebaceous appendages. Reactivity scores were based on the degree of several factors: Polymorphonuclear cells, lymphocytes, macrophages, giant cells, necrosis, neovascularization, fatty infiltrate, edema, hemorrhage, mineralization, dilated blood vessels, crust, and hyperkeratosis.

### Planimetric Evaluation and Photographs

The outlines of the wound edge and areas covered with slough/eschar, hard crusts, granulation tissue, or epithelium were drawn on transparent sheets on the listed days (Figure 1D). The sheets were scanned, and an algorithm was developed to automatically measure the area of the wound. The photographs were taken with a digital camera in a standardized manner, with a flash, at a fixed distance. Square metal boxes were used to calibrate the distance.

### Cultivation and Differentiation of Monocyte to Macrophage Cells

THP-1 monocyte cell line (ATCC, TIB-202) was cultured in RPMI (Lonza, 12-702F) supplemented with 10% fetal bovine serum (ATCC, 30-2025) and 1% Penicillin-Streptomycin (Sigma Aldrich, P4333). This is from now on called “medium”. Cells were kept at 37℃, 5% CO_2_, humidified atmosphere. For differentiation of THP-1 into M0 and M1, the protocol used was essentially as described in Baxter et al^23^: In short, cells were seeded at a concentration of 2 × 10^5^ cells/ml in 24 well plates and exposed to Phorbol 12-muristate 13-acetate (PMA, 200 nM, Sigma-Aldrich, P8139) for 24 hours. This transformed the cells from monocyte in suspension to adherent macrophages (M0). Medium was removed from M0 cells and replaced by medium containing interferon (IFN)-γ (20 ng/ml, Sigma-Aldrich, GF305) and Lipopolysaccharide (LPS, 250 ng/ml, Sigma-Aldrich, P4391) and incubated for 24 hours causing an M0 to M1 differentiation. Collex (ca 200 mg) and Collex without HTX (ca 200 mg) were extracted in medium (1 ml) and incubated at 37℃ for 4 hours. This causes a release of the HTX content from Collex into the medium at a protein concentration similar to 2% HTX. In parallel, HTX (2%) and IL-4 (40 ng/ml) were prepared. Medium containing IFNγ and LPS was replaced by medium containing Collex extract, Collex without HTX extract, HTX, IL-4 or medium only and incubated for an additional 48 hours. Supernatant from these cells were tested for levels of interleukin (IL)-1β, and the cells were tested for cell death and oxidative stress. An outline of the method is illustrated in Figure 1E.

### Interleukin-1β ELISA Assay

The level of IL-1β was determined from the medium of the cell cultures by Human IL-1 beta ELISA Kit (Abcam, ab214025) according to the manufacturer’s recommendations.

### Propidium Iodide Staining

Propidium iodide (PI) (Sigma Aldrich, P4170), a fluorescent DNA-binding dye, was used to determine cell death. It acts by penetrating the cell membranes of dead and dying cells. Forty-eight hours after HTX, Collex or Collex without HTX treatment, cells were detached by scraping and pipetting to obtain single cells and transferred to Eppendorf tubes on ice. PI was added to the tubes (final concentration 1 mg/ml), and the signal was analyzed immediately by flow cytometry (MACSQuant Analyzer 10 Flow Cytometer; Miltenyi Biotec) by excitation at λ = 488 nm and detection at λ = 585/40 nm.

### Reactive oxygen species staining

CellROX green (Thermo Fisher, C10492) was used for the detection of Reactive oxygen species (ROS), and is a dye converted to fluorescent molecules upon intracellular oxidation. 48 hours after HTX, Collex or Collex without HTX treatment, cells were detached by scraping and pipetting to obtain single cells and transferred to Eppendorf tubes. CellROX green was added to the tubes (final concentration 5 μM), and the tubes were incubated at 37°C for 30 minutes. The signal intensity was analyzed by flow cytometry (MACSQuant Analyzer 10 Flow Cytometer; Miltenyi Biotec) by excitation at λ = 488 nm and detection at λ = 525/50 nm.

### Statistical Analysis

All statistical analysis was performed using GraphPad Prism software, version 9.4 (GraphPad Software, La Jolla, CA). The significance between the treatment groups were determined by an unpaired *T*-test and statistical significance was defined as *P* value <.05. In the animal experiment, planimetric evaluation from Day 6 was excluded from the analysis. The mean area of the wounds showed the same trend (smallest area with Collex treatment, highest area with Jelonet treatment) however, the planimetric drawings were performed by a different veterinarian and showed an overall markedly reduced wound area of all wounds compared to the other days. Except planimetry, other data from Day 6 are included. In addition, two outlier wounds were identified (one from Collex and one from Jelonet treatment) and removed from the analysis. One outlier appeared substantially more burned than the other 47 wounds, and the other outlier was subjected to an erroneous drawing of the wound border at planimetric evaluation.

## RESULTS

### Sterility and Biocompatibility of Collex

Before testing Collex on animals, the sterility of the end products was evaluated on five Collex dressings using PyroGene™ endotoxin analysis. The endotoxin levels were below 0.005 EU/ml for all five Collex samples analyzed, well below the limits for medical devices (0.5 EU/ml, according to the Center for Devices and Radiological Health and European Pharmacopeia).

**Figure 2.**
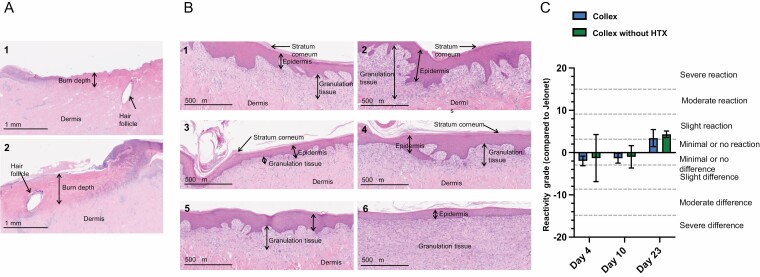
Burn depth and reactivity. (A) and (B) hematoxylin and eosin-stained biopsy slides of wounds at Days 4 (A) and 23 (B). (A) Burn depth as revealed by denaturation of dermal collagen (deeper eosinophilic staining). (B) Two representative wounds, as determined by evaluating pathologist, from each treatment at the end of the experiment: 1 and 2: Collex, 3 and 4: Collex without HTX, 5 and 6: Jelonet. (C) Scores on reactivity of Collex and Collex without HTX compared to Jelonet, determined by pathologist at Scantox A/S. Score 0.0–2.9: no reactivity, 3.0–8.9: slight rection, 9–15.0: moderate reaction, >15.1: severe reaction. Data presented are mean ± SEM, N = 3 (Days 4 and 10) and N = 9 (Day 23). Blue bars represent reactivity in wounds treated with Collex and green bars represent reactivity in wounds treated with Collex without HTX.

Preclinically safety and efficacy of Collex were tested on thermal burn wounds generated on the back of Göttingen minipigs (methodology illustrated in Figure 1A–D). The wound depth was estimated from a microscopic evaluation of hematoxylin and eosin-stained slides of biopsies from the wounds on Day 4. Two independent wounds illustrating wound depth is shown in Figure 2A. Burn depth is visualized by deeper eosinophilic staining of denatured dermal collagen and extends to the hair follicles.

To assess the local effect on skin reactivity, hematoxylin and eosin-stained biopsy slides were evaluated. Figure 2B shows two representative wounds from each treatment at Day 23. The photos illustrate a complete epidermis on top of granulation tissue and low reactivity. Based on a semi-quantitative scoring system, the reactivity of Collex and Collex without HTX compared to Jelonet was evaluated (Figure 2C). On Days 4 and 10, no to slight negative reactivity was detected. On Day 23, Collex showed a slight reactivity compared to Jelonet (mean value 3.4, Figure 2C). Furthermore, according to macroscopic assessment by veterinarians at Scantox A/S, there was no apparent infection in any of the wounds (unpublished data).

### The Effect of Collex on Healing of Porcine Burn Wounds

**Figure 3.**
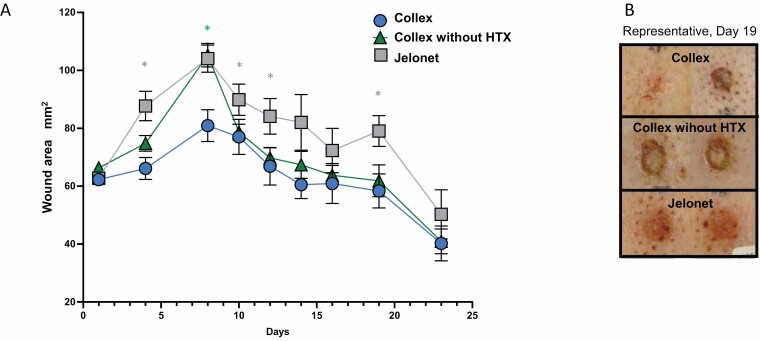
Wound area during the experiment. (A) Development of wound area from all wounds calculated from the planimetric drawings. Data presented are the mean values ± SEM. Because wounds are terminated after biopsy at Days 4 and 10, number of wounds are not the same for all days: N = 15 (Days 1 and 4), N = 12 (Days 8 and 10) and N = 9 (Days 12 to 23). Blue circles represent area of wounds treated with Collex, green triangles represent area of wound treated with Collex without HTX, and gray squares represent area of wound treated with Jelonet. Gray star indicates *P* < .05 between Collex and Jelonet. Green star indicates *P* < .05 between Collex and Collex without HTX. Significance is determined by unpaired *T*-test. (B) Photos of two representative burn wounds from each treatment, Day 19. Area of the wounds presented were closest to the mean area. Wound areas of the representative wounds: Collex: 65 mm^2^ and 52 mm^2^ (mean: 58 mm^2^), Collex without HTX: 63 mm^2^ and 62 mm^2^ (mean: 62 mm^2^), and Jelonet: 72 mm^2^ and 81 mm^2^ (mean: 79 mm^2^).

During dressing changes, each wound was evaluated macroscopically by a veterinarian at Scantox A/S which reported the formation of hard crust on wounds treated with Jelonet on Days 10, 12, and 14, but not on the wounds treated with Collex or Collex without HTX (unpublished data). To assess the wound size over time, the wound area was calculated from the planimetric drawings (Figure 3A). The average wound size initially increased for the first week before it gradually decreased. From Days 1 to 4, wounds treated with Collex increased in size by 8%, whereas wounds treated with Collex without HTX and Jelonet increased by 13% and 40% respectively (Figure 3A). Furthermore, from Days 1 to 8, wounds treated with Collex increased in size by 42%, whereas wounds treated with Collex without HTX and Jelonet increased in size by 59% and 66% respectively. At all timepoints tested, wounds treated with Collex had a lower average area compared to wounds treated with Jelonet with statistically relevant differences at Days 4, 10, 12, and 19 (*P* < .05), In addition, the area of wounds treated with Collex was lower than the area of wounds treated with Collex without HTX at all time points except Day 23 where histopathological evaluation indicated that wounds were fully healed. However, a statistically significant difference was only observed on Day 8 (green star in Figure 3A, *P* < .05).

For visual illustrative purposes, digital photos were captured in a standardized manner. Figure 3B shows two representative wounds from each treatment at Day 19 when the wounds were close to being fully healed. The wounds were chosen based on the planimetric evaluation and had the areas closest to the mean area in the respective treatment category. The photos visualize a reduced wound area and reduced rubor at the wound edge and the surrounding tissue in wounds treated with Collex compared to the other treatments.

### The Effect of Collex on Inflammation in Porcine Burn Wounds

**Figure 4.**
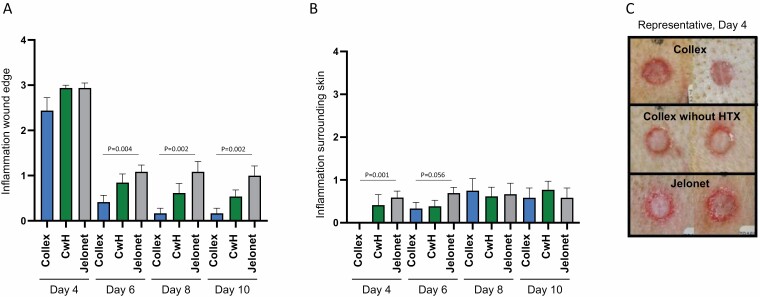
Burn-induced inflammation. Inflammation scoring of the (A) wound edge and (B) surrounding skin based on macroscopic evaluation by Scantox A/S. (A and B) Grading system: 0: not present, 1: minimal, 2: slight, 3: moderate, 4: marked. Because wounds are terminated after biopsy at Days 4 and 10, number of wounds are not the same for all days: N = 15 (Day 4) and N = 12 (Days 6 and 8). Data shown are mean values ± SEM. *P* values are determined by unpaired *T*-test. (C) Photos of two representative burn wounds from each treatment, Day 4. The wounds are the same as shown in Figure 3B.

In the macroscopic evaluation, inflammation was assessed by veterinarians at Scantox A/S and given a score from 0 (not present) to 4 (marked) (Figure 4A). On Day 4, inflammation at the wound edge was evaluated to be 17% lower for wounds treated with Collex, compared both to wounds treated with Collex without HTX and with Jelonet. On Day 6, overall inflammation was reduced, but was markedly lower in wounds treated with Collex compared to wounds treated with Collex without HTX and Jelonet with 51% and 62% difference to Collex respectively. On Days 8 and 10, the level of inflammation was essentially similar to Day 6, with wounds treated with Collex displaying the lowest levels of inflammation. Inflammation in the surrounding skin was low (Figure 4B); however, at Days 4 and 6, a significant reduction of inflammation was seen around wounds treated with Collex compared to wounds treated with Jelonet. On Days 8 and 10, there were still low levels of inflammation in the surrounding skin of all wounds, and no differences between the treatments were observed.

Inflammation was also evaluated by histopathologic assessment of hematoxylin and eosin-stained biopsy slides of wounds from Days 4 and 10. This analysis indicated a slightly reduced inflammatory response in the wounds treated with Collex compared to wounds treated with Collex without HTX and Jelonet (unpublished data). However, with a small sample size (N = 3) and large variations, statistically significant values were not obtained. Photos of representative wounds from Day 4 visualize reduced redness at the wound edge in wounds treated with Collex compared to Collex without HTX and Jelonet (Figure 4C). The wounds depicted are the same wounds as shown in Figure 3B.

### The Effect of Collex and HTX on Pro-inflammatory M1 Macrophages

**Figure 5.**
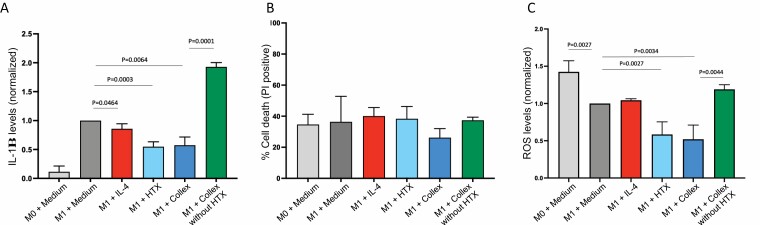
M1-polarized macrophages in vitro. (B) IL-1β secreted from macrophages activated according to (A), detected by ELISA assay. The data presented are normalized against the level secreted from cells treated with PMA, IFNγ and LPS (M1 + Medium). (C) Cell death as determined by PI-positive cells. Cells are from the same experiment as (B). (D) ROS levels determined by CellROX measured on Flow cytometry. The data presented are normalized against the ROS level in cells treated with PMA, IFNγ and LPS (M1 + Medium). Cells are from the same experiment as (B and C). (B, C, and D) Data presented are mean values ±SD, N = 2 (PMA treatment only, M0) and N = 3 (M1). *P* values are determined by unpaired *T*-test.

To assess the anti-inflammatory response in more detail, a human monocyte cell line (THP-1) was differentiated into pro-inflammatory M1 macrophages, treated with: medium exposed to Collex; medium exposed to Collex without HTX; HTX; interleukin 4 (IL-4) and the level of secreted IL-1β analyzed by ELISA (methodology illustrated in Figure 1E). Differentiation of cells from M0 (PMA only) to M1 (PMA + IFNγ + LPS) increased the level of IL-1β (Figure 5A). Notably, treatment with both Collex and HTX reduced secretion of IL-1β, by 45% and 43% respectively. Collex without HTX, however, did not cause reduced levels, but rather enhanced secretion of IL-1β. Treatment of M1 cells with the know IL-1β inhibitor IL-4 slightly reduced secretion of IL-1β (14%). To assess cell survival, cells from the same experiment were collected, stained with the cell death dye propidium iodide (PI) and analyzed by flow cytometry. As shown in Figure 5B, neither HTX nor Collex caused an increase in cell death. To address the potential antioxidant activity of HTX, the same M1-polarized macrophages were stained with the ROS dye CellROX and analyzed by flow cytometry. As shown in Figure 5D, the ROS levels were reduced in cells treated with both Collex and HTX, with 48% and 41% reduction respectively. Treatment with IL-4 or Collex without HTX did not cause reduced levels of ROS.

## DISCUSSION

Results from this study suggest that Collex is sterile, biocompatible, and able to accelerate the healing of second-degree burns in minipigs. HTX contains proteins and lipids and is sensitive to oxidation and excessive heat. Therefore, conventional sterilization used on medical devices such as exposure to ethylene oxide or autoclavation could not be used on Collex.^24^ Instead, sterile ingredients were sourced, and the production was finalized by an aseptic procedure. Endotoxins give an estimate not only for the sterility of the product at the time of testing, but also for whether there has been bacterial contamination present during the production chain.^25^ Using endotoxin analysis to assess the sterility therefore assured that both Collex and its production process were sterile.

Histopathological examination from Day 4 biopsies suggested that wounds inflicted were partial thickness burn wounds, because denatured collagen (deeper eosinophilic staining) extended to the hair follicles. Partial-thickness burn wounds in humans typically heal within 14 days.^26^ It was therefore surprising that the wounds were not fully healed even at Day 23, according to planimetric evaluation. However, histopathologic evaluation at Day 23 showed that all wounds, except one wound treated with Jelonet, were fully re-epithelialized at Day 23. Therefore, the wounds were probably fully healed towards the end of the study, with a thin epithelial layer covering the wounds. Alternatively, the burn wounds were deeper than anticipated. Nevertheless, the wounds at Day 1 were all equally deep, and assessing the safety and efficacy of Collex was our primary objective. Collex is a medical device and its biocompatibility needs to be addressed. In that respect, the local effect on skin reactivity was analyzed. Because Jelonet is used on burn wounds in hospitals, wounds treated with Jelonet was used as a control. A slightly increased reactivity was detected for Collex at Day 23. Although the value was in the lower end of the category of slight reactivity, minor changes to Collex may be considered: Calcium, which is used in Collex for crosslinking of alginate fibers, is beneficial for wound healing.^27^ However, high concentrations may cause skin reactivity and cell toxicity.^28,29^ Unpublished data from our lab indicate that Collex produced with lower calcium concentrations cause markedly reduced cytotoxicity in sensitive cells. Hence, Collex with lower levels of calcium may be considered for future clinical testing.

Compared to Jelonet, planimetric evaluation showed that wounds treated with Collex had a faster rate of healing throughout the study. Compared to both Jelonet and Collex without HTX, a pronounced divergence between the treatments appeared in the initial post-injury phase. Partial-thickness burn wounds suffer from secondary necrosis for days after the actual trauma due to excessive inflammation. During the first week of healing, the reduced degree of secondary necrosis observed in Collex-treated wounds indicated that Collex reduced the inflammatory response. Although not statistically significant, histopathologic evaluation from Days 4 and 10 agrees with an anti-inflammatory effect of Collex. In addition, macroscopic evaluation, as well as photos from Day 4, further support an anti-inflammatory effect of Collex. Together, these observations strongly indicate that dermal treatment with Collex reduced the burn-induced inflammatory response.

**Figure 6.**
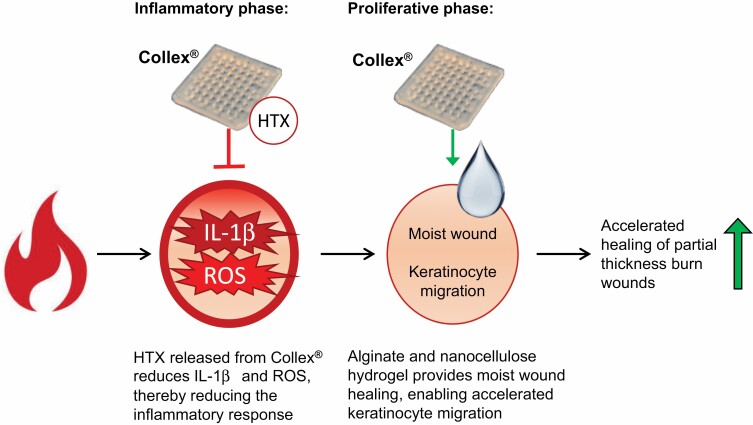
Model describing a hypothetical mode of action for Collex. Burn wounds are heavily inflamed and have abundant secretion of pro-inflammatory IL-1β and excessive generation of ROS. Our data suggest that Collex (containing HTX) dampens the inflammatory response in vivo, possibly by reducing the level of the pro-inflammatory cytokine IL-1β and reducing the level of ROS. Both in the inflammatory and the proceeding proliferative phase of wound healing, Collex appears to accelerate the wound closure by ensuring a moist, protected environment.

Burn wounds cause excessive inflammation and ROS, tightly associated with increased apoptosis and necrosis.^3,4,30,31^ The excessive inflammation is partly caused by reduced polarization of pro-inflammatory M1 macrophages into anti-inflammatory M2 macrophages.^32^ Treating M0 macrophages with LPS and IFNγ transforms M0 macrophages into M1 macrophages, secreting increased levels of the pro-inflammatory cytokine IL-1β, mimicking the in vivo situation.^2,23,32^ Our in vitro data on pro-inflammatory human macrophages confirm an anti-inflammatory effect of Collex as suggested by results from the animal study. In addition, results on intracellular ROS suggest an antioxidant effect of Collex. Stock HTX at a concentration of 2% (v/v) results in a similar HTX concentration as HTX extracted from Collex. Given that stock HTX and Collex reduced the levels of IL-1β and ROS to similar levels, while Collex without HTX did not, it is highly likely that the anti-inflammatory and antioxidant effect of Collex is caused by HTX. Based on our findings, a hypothetical model for a mode of action for Collex is presented in Figure 6. Collex, aided by HTX, appears to suppress the burn-induced inflammation by reducing the level of IL-1β and ROS. In the proceeding proliferative phase of burn wound healing, Collex provides a moist environment that is favorable for keratinocyte migration.^33^ It is likely that these two mechanisms of Collex combined contributes to an accelerated wound healing of partial thickness burn wounds.

Among current dressings indicated for partial thickness burn wounds, there are some common properties that are generally regarded as important: The dressing should cover and protect the wound, the dressing should be absorbent to manage exudates, and it should be moist to accelerate re-epithelization. These are all mechanical properties important to facilitate an optimal environment for wound closure and for reducing the risk of infection during healing.^1,34^ In addition, several dressings intended for partial thickness burn wounds also contain silver, such as Mepilex^®^ Ag or Aquacel^®^ Ag Burn.^9^ Silver ions (Ag^+^) released from these dressing have antibacterial activity,^35,36^ however, studies have also shown that high release of Ag^+^ is correlated with cytotoxicity, and may cause histological damage and delayed healing.^35,37^

Collex shares the common mechanical properties crucial for the dressing’s wound healing properties, and it is the authors’ hypothesis that collectively, they make up the primary mode of action. Results from the planimetric evaluation support this, where both Collex and Collex without HTX showed reduced wound areas compared to Jelonet, with less pronounced differences between the two. The ability of Collex to keep the wound bed moist is supported by the macroscopic evaluation, where hard crusts were identified on wounds treated with Jelonet, but not on any of the wounds treated with Collex or Collex without HTX. The link between moist wound healing and prevention of crust formation and enhanced rate of re-epithelialization is well established.^33,38^ The anti-inflammatory and antioxidant actions shown in this study thus appear to be ancillary modes of action of Collex. However, these properties remain important, because they fulfill an unmet medical need in wound management, and are not typically found in dressings in regular use for burn management today.^9,18,39^ Excessive ROS is known to activate the inflammatory response and vice versa, hence reducing ROS may by itself reduce inflammation.^40^ Given the detrimental effects of ROS and inflammation on wound progression and scarring,^41,42^ it is not surprising that development of novel burn care therapies focus on both reduction of the inflammatory response^2,3,43^ and reduction of ROS levels.^4,8,16,44,45^ The anti-inflammatory action of Collex, which reduces secondary necrosis, appears to give wounds treated with Collex about a week’s head start in terms of wound closure compared to wounds treated with Jelonet. If this could result in faster healing of severe burns, the duration of treatment might be reduced, and patient management might more rapidly be transferred to lower tiers of care, for example from intensive care through inpatient, outpatient, and self-care.

The antioxidant properties of Collex and HTX indicated by this study do not come as surprise given the natural composition of the salmon roe-derived HTX. The components of HTX make up a cocktail of chemical substances with known anti-inflammatory and antioxidant properties: Unsaturated fatty acids with marine origin have previously proven to be anti-inflammatory and are, because of this, suggested to improve several aspects of human health.^46^ Of note, Omega-3 and Omega-6 found in fish oil are reported to be important in the antioxidant defense.^47^ Zinc is the most abundant mineral found in HTX (unpublished data). Zinc has been used in wound healing for many years and improves the healing through several mechanisms where anti-inflammation and antioxidant effects are significant.^48–51^ Lastly, HTX is rich in the lipoprotein Vitellogenin (unpublished data). Vitellogenin is the major egg yolk precursor protein that provides protein- and lipid-rich nutrients for the developing fish embryo. Furthermore, it is reported to act as an antioxidant, protecting the host from oxidative stress.^19^ It has not been established which of these components gives HTX its anti-inflammatory and antioxidant effects. However, based on previous unpublished studies from our lab, the effects appear to come from a combination of several of the components, rather than one in isolation.

### Study Limitations

Some potential limitations present in our study must be acknowledged. For practical reasons, wound assessment, macroscopic and microscopic, was performed unblinded. This may cause a risk of biased interpretation; however, assessments were good laboratory practice (GLP) compliant and performed by independent veterinarians or pathologists at Scantox A/S. For macroscopic evaluation, the assessment was performed during dressing changes. For microscopic evaluation, blinded examination is not recommended as it increases the risk of missing subtle treatment-related changes.^52^ The porcine model was chosen based on its similarity to humans, however, there are some obvious differences: The pig skin is thicker, lacks eccrine glands, and its immune system is slightly different than humans. In the study design, we chose to make many small wounds instead of fewer large wounds. Differences in wound size may have been clearer in larger wounds. In addition, more biopsies could have been collected from the wound edge during the course of healing without terminating the wounds. It would for instance have been of interest to have the histological evaluation of all 16 wounds on Days 4 and 10, and not only three, which were too low to obtain significant values. However, larger wounds could result in animal suffering, would limit the number of wounds, and would limit the freedom to randomize the localization of the wounds.

### Conclusion

Data from our study show that Collex improved the healing of partial-thickness burn wounds, particularly in the initial inflammatory phase. Evaluating Collex with and without HTX, the unfertilized salmon roe extract, the shared primary mode of action appeared to be mechanical cover-and-protect properties combined with a moist environment created by the alginate and nanocellulose hydrogel. In addition, as indicated in the minipigs and supported by macrophage experiments, we found that roe extract was responsible for an apparent ancillary anti-inflammatory and antioxidant effect. This study has implications for the potential use of unfertilized salmon roe in advanced wound care, including bioactive wound dressings. More research is needed to document the exact mechanisms and raw materials responsible for the apparent biological effects. For the authors, this study has further triggered a plan for clinical testing of Collex with roe extract. We believe that Collex has the potential to improve the treatment of partial-thickness burn wounds, particularly during the initial inflammatory phase, resulting in better burn care for patients and cost savings for care providers.

## Notes

*Funding*: This work has been supported by ERA-NET Cofound on the Blue Bioeconomy (ID: 151), Innovation Fund Denmark, The Research Council of Norway, and Formas Sweden.
